# Presumed Idiopathic Replacement Resorption in a Permanent First Molar in a Child With Multiple Tooth Agenesis: A Case Report

**DOI:** 10.7759/cureus.108533

**Published:** 2026-05-09

**Authors:** Cristina Coralia Nistor, Anca Oana Dragomirescu, Ioana Suciu

**Affiliations:** 1 Endodontics, Faculty of Dentistry, “Carol Davila” University of Medicine and Pharmacy, Bucharest, ROU; 2 Orthodontics and Dentofacial Orthopedics, Faculty of Dentistry, “Carol Davila” University of Medicine and Pharmacy, Bucharest, ROU

**Keywords:** bioceramic, cone-beam computed tomography, endodontics, permanent molar, replacement resorption, tooth agenesis

## Abstract

Replacement resorption is a rare pathological condition in permanent teeth, characterized by progressive substitution of root dentin with bone tissue, typically following traumatic injuries. This case report presents an unusual occurrence of replacement resorption in the mesial root of the mandibular right first permanent molar (46) in an eight-year-old boy with no documented history of trauma, associated with non-syndromic agenesis of multiple permanent teeth (15, 34, 35, 44, 45). Despite the reserved long-term prognosis, conservative endodontic management was pursued due to the significant tooth agenesis. This case highlights the importance of advanced imaging in diagnosis, the challenges of managing replacement resorption, and the potential for successful conservative treatment when appropriate protocols are followed.

## Introduction

External replacement resorption is a pathological condition, also termed ankylosis, characterized by the replacement of normal dental tissues with bone [1]. This occurs because of etiologic factors acting upon the protective layer of the root and the periodontal ligament [2]. Among these factors are traumatic forces, including orthodontic forces, as well as local metabolic disturbances within the periodontal ligament (periodontal inflammation) [3,4]. Replacement resorption is most associated with severe dental trauma, particularly tooth avulsion, intrusive luxation, and replantation procedures performed after extended extra-alveolar time [5]. Histologically, ankylosed areas show continuity between bone and root dentin, with the complete absence of the periodontal ligament space [6].

Anterior teeth are more frequently affected than posterior teeth, given their greater susceptibility to traumatic injury [7]. Replacement resorption has clinical significance in growing children, where the ankylosed tooth may later become infraoccluded as surrounding teeth continue to erupt and alveolar bone develops vertically [8]. This progressive infraocclusion leads to functional concerns, tilting of adjacent teeth, and localized arrest of alveolar bone growth [9].

The classic diagnosis for this condition is primarily radiographic [10]. The appearance shows interruption of the periodontal ligament and the disappearance of radicular dental tissues in various areas [10]. Rarely can this be visualized with precision on conventional radiographs [11]. Cone-beam computed tomography (CBCT) is the imaging modality of choice for this purpose, providing detection and three-dimensional visualization of the extent and location of resorption defects that may be missed on conventional radiographs [12].

This case is unusual because replacement resorption occurred in a mandibular first molar without trauma, in a child with five missing permanent teeth, and was initially suspected only after apex locator discrepancies led to CBCT.

This clinical case illustrates the diagnostic challenges encountered, the critical role of CBCT in treatment planning, and the successful conservative management approach employed despite the guarded long-term prognosis.

## Case presentation

An eight-year-old male patient was referred to an endodontic specialist for the evaluation and management of the mandibular right first molar (tooth 46). The patient was medically healthy (ASA I physical status classification), with no known drug allergies, systemic conditions, or current medications [13]. Notably, the child's father, present during the consultation, had a surgically corrected lip and cleft palate (palatoschisis) and exhibited residual speech distortion.

The patient reported experiencing acute symptoms one week prior, including mild pain and tenderness in the right mandibular first molar region. Emergency intervention had been performed by a general dentist, involving access cavity preparation and placement of a temporary filling. The patient was subsequently referred for definitive endodontic treatment.

At the time of presentation, clinical examination revealed no facial asymmetry or systemic symptoms. The patient appeared well-developed and cooperative during examination. Intraoral inspection showed extensive coronal caries associated with tooth 46. The surrounding mucosa appeared normal, with no evidence of sinus tract formation, soft tissue swelling, or significant periodontal involvement. Tooth 46 was found to be non-responsive to cold testing (Endo-Ice, Coltène/Whaledent, Altstätten, Switzerland), indicating pulpal necrosis. The tooth did not exhibit tenderness to percussion or palpation.

A panoramic radiograph (Figure 1), obtained prior to the emergency treatment, revealed a significant finding of non-syndromic agenesis of five permanent teeth: the upper right second premolar (tooth 15), the left mandibular first and second premolars (teeth 34 and 35), and the right mandibular first and second premolars (teeth 44 and 45).

**Figure 1:**
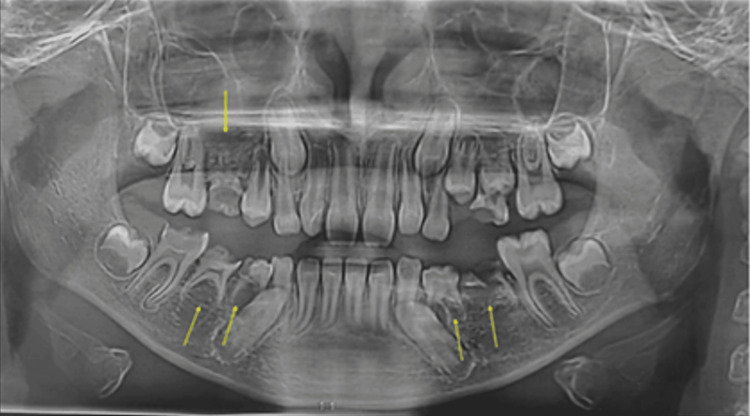
Preoperative panoramic radiograph highlighting agenesis of five permanent teeth (15, 34, 35, 44, and 45)

The periapical radiograph (Digora, Soredex, Tuusula, Finland) demonstrated tooth 46 with extensive coronal caries extending to the pulp chamber, a coronal temporary filling, and incomplete formation of both mesial and distal root apices with divergent configuration (Figure 2). A more pronounced radiolucency was observed in relation to the mesial root, which also showed a tendency toward double curvature in the apical third.

**Figure 2:**
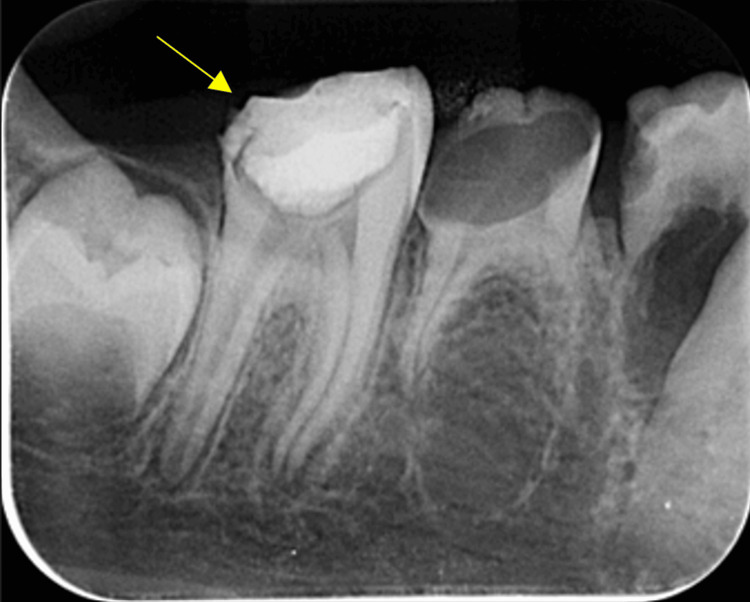
Preoperative periapical radiograph revealing extensive coronal caries of tooth 46 with coronal temporary filling material visible

Based on clinical and radiographic findings, the initial diagnosis was pulp necrosis with asymptomatic apical periodontitis affecting tooth 46. Nonsurgical root canal treatment was considered as the conservative treatment approach of choice. Written informed consent was obtained from the patient's father after detailed discussion of the treatment plan, potential complications, and prognosis.

After rubber dam isolation and administration of local anesthesia, the temporary filling was removed. A pre-endodontic coronal build-up was performed using composite adhesive materials (Charisma, Kulzer GmbH, Hanau, Germany) to provide adequate isolation and structural support for the rubber dam clamp. Under magnification (dental operating microscope), three root canal orifices were identified and confirmed: distal, mesiobuccal, and mesiolingual.

Working length determination for the distal canal was accomplished using an electronic apex locator (Root ZX II, Morita Corporation, Kyoto, Japan) and was established at 21.5 mm. However, significant difficulties arose when attempting to determine the working length for the mesial canals. Despite taking appropriate precautions to avoid errors commonly encountered in multi-rooted teeth with immature apices - including ensuring that sodium hypochlorite solution was not present in the pulp chamber, avoiding flooding the canals with irrigant, and selecting files well-adapted to the canal diameter - the apex locator consistently indicated proximity to the apical periodontium when the file was positioned only at the estimated middle third of the canal based on radiographic assessment.

This apex locator reading suggested a potential communication between the root canal system and the periodontal tissues at an unexpected location along the root length, raising suspicion for root perforation, resorptive defect, or anatomical anomaly. Given these findings and the potential for iatrogenic complications, the treatment was postponed. Calcium hydroxide paste (UltraCal XS, Ultradent Products, South Jordan, UT, USA) was placed superficially in the canal orifices as a temporary intracanal medicament. The patient's father was informed of the necessity for CBCT examination to clarify the anatomical situation before proceeding with definitive treatment.

CBCT images were acquired using Veraviewepocs 3D P (R100) equipment (J. Morita MFG Corp., Kyoto, Japan) with standardized scan parameters: 90 kV, 3 mA, 9.4 seconds exposure time, 125 μm spatial resolution (voxel size), and a 40×40 mm field of view (FoV) centered on the area of interest. The small field of view was selected to minimize radiation exposure while providing adequate diagnostic information for the region of concern.

CBCT analysis revealed a replacement resorption defect in the middle third of the mesial root of tooth 46 (Figure 3a). The resorptive lesion was located between the two mesial canals (mesiobuccal and mesiolingual), extending toward the furcation area (Figure 3b). Three-dimensional measurements indicated that the defect extended across a coronal-apical length of 6.06 mm (Figure 3c). The CBCT images demonstrated characteristic features of replacement resorption, including obliteration of the periodontal ligament space in the affected area and replacement of root dentin with bone-density tissue. The axial sections clearly showed the location of the defect between the two mesial canals, while sagittal sections demonstrated its vertical extent (Figures 3a, 3c).

**Figure 3:**
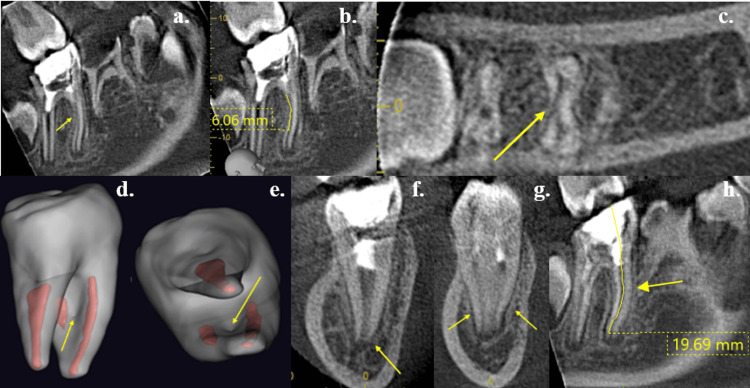
(a) CBCT sagittal section showing resorption area in the middle third of the mesial root. (b) CBCT sagittal section on which the dimension of the resorption area of the mesial root was measured. (c) Axial CBCT section showing resorption of the mesial root in the furcation area. (d, e) Automatic segmentation of tooth 46 using Diagnocat, showing the area of bone tissue invasion into the mesial root (root canals are depicted in red). (f) Coronal CBCT section through the distal root showing the divergent, immature apex. (g) Coronal CBCT section through the mesial root showing the double-curved mesial canals. (h) Working length estimation of the mesial canals on the sagittal CBCT section. CBCT, cone-beam computed tomography

Automatic segmentation utilizing the Diagnocat software (Diagnocat Inc., San Francisco, CA, USA) enabled enhanced visualization of the divergent, immature root apices and the resorptive defect, which manifested as a marked concavity on the distal surface of the mesial root at the middle third level, oriented toward the furcation area (Figures 3d, 3e).

Following review of the CBCT diagnosis, a comprehensive discussion was held with the patient's father regarding the etiology, prognosis, and treatment options. Importantly, the father did not recall any traumatic event involving the patient, including falls, sports injuries, dental trauma, or other incidents that might explain the development of replacement resorption.

Therapeutic options were thoroughly explained, including nonsurgical root canal treatment with close monitoring, extraction and space maintenance pending future prosthetic rehabilitation, or extraction with orthodontic space closure, if feasible.

The extremely reserved long-term prognosis for replacement resorption was discussed, including the high risk of progressive infraocclusion during the patient's remaining growth years, potential for continued resorption leading to tooth loss, and possible complications during endodontic treatment. Despite the guarded prognosis, the father insisted on conservative management and preservation of the tooth. His decision was strongly influenced by the significant agenesis of permanent teeth, which made preservation of every existing tooth particularly important for maintaining occlusal function and future treatment options.

In the second treatment session, following administration of local anesthesia and rubber dam isolation, mechanical instrumentation of the distal canal was completed using ROTATE instruments (VDW, Munich, Germany) with 25/0.06 taper configuration. The canal was prepared to the previously established working length of 21.5 mm (Figure 3f).

For the mesial canals (Figures 3g, 3h), a conservative instrumentation approach was employed. The canals were accessed along the estimated working length determined by CBCT analysis and radiographic correlation using ROTATE instruments sized 25/0.06. The CBCT-derived working length was confirmed radiographically (Figure 4). Given the proximity of the replacement resorption defect to the mesial canals, minimal shaping was performed to avoid perforation into the resorptive area. The instrumentation focused on debridement and removal of necrotic tissue while preserving maximum dentin thickness.

**Figure 4:**
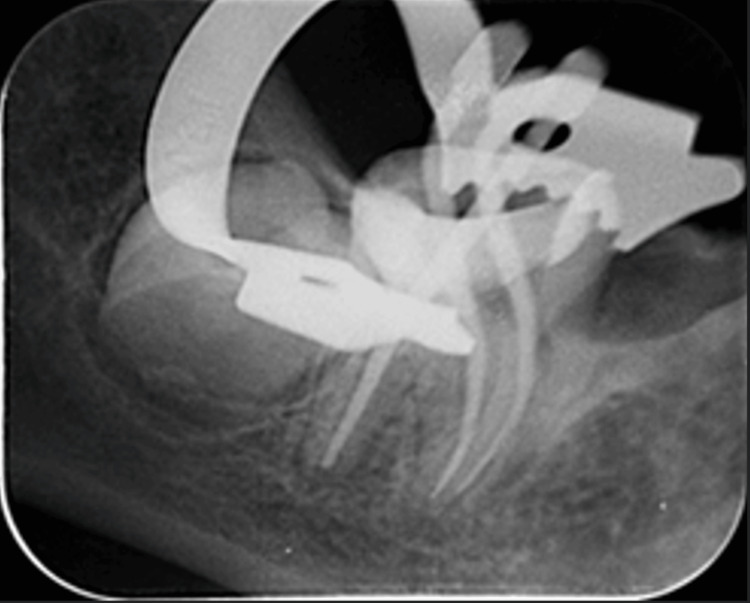
Periapical radiograph confirming the working length

All canals were gently irrigated using 2% sodium hypochlorite solution (Chloraxid, Cerkamed, Stalowa Wola, Poland). The solution was delivered using negative pressure irrigation with the EndoVac system (Kerr Corporation, Orange, CA, USA).

The obturation technique was selected based on the anatomical challenges presented by the case. The distal canal was obturated using a dual technique: an apical plug of bioceramic putty (Well-Root PT, Vericom, Gyeonggi-do, South Korea) approximately 3-4 mm in length was placed at the apex, followed by placement of resin-based sealer (AH Plus, Dentsply Sirona, Charlotte, NC, USA) and warm vertical compaction of gutta-percha.

The mesial canals presented challenges due to the double curvatures visible in both sagittal and frontal planes on CBCT, minimal canal shaping to preserve dentin integrity, and proximity to the replacement resorption defect. To address these challenges, the mesial canals were obturated entirely with bioceramic putty (Well-Root PT, Vericom). The placement technique required adaptation of conventional methods: a ROTATE instrument with 0.25-mm diameter and 4% taper was selected, and the tip was carefully cut using a high-speed diamond bur to create a thin, flexible plugger. This modified instrument was used to compact the bioceramic putty incrementally from the apical foramen coronally, ensuring complete obturation without voids or gaps, particularly in the area adjacent to the resorptive defect, where adequate sealing was critical.

A control radiograph was obtained immediately following obturation to confirm adequate fill of all canals and proper material adaptation. The radiograph demonstrated complete obturation of the distal canal with the hybrid technique and homogeneous fill of both mesial canals with bioceramic material extending to the radiographic apex (Figure 5).

**Figure 5:**
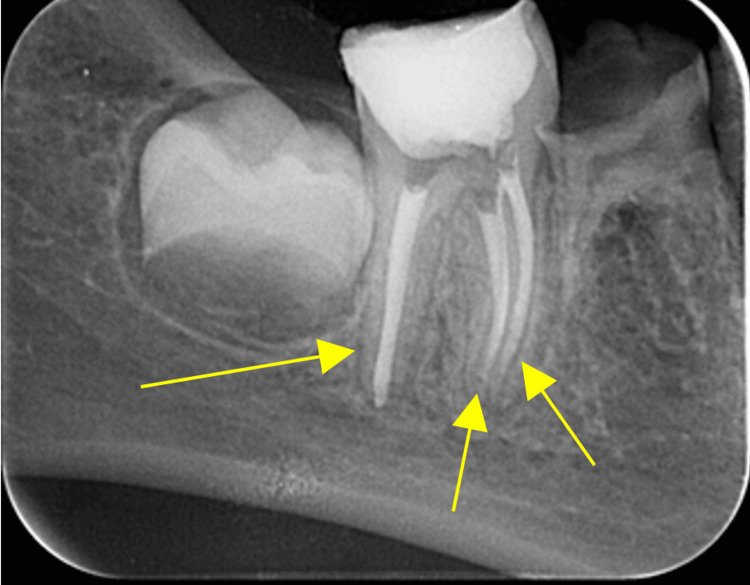
Postoperative periapical radiograph of tooth 46 with complete obturation of distal and mesial canals

In a subsequent appointment, the temporary restoration was removed, and the access cavity was cleaned. A definitive adhesive coronal restoration was placed using composite resin material.

The patient was scheduled for follow-up evaluation after six months but returned after one year due to scheduling conflicts. Clinical examination revealed the tooth to be completely asymptomatic and functional, with no reported symptoms of pain, sensitivity, or discomfort. Importantly, no clinical signs of infraocclusion were evident, with tooth 46 maintaining its occlusal position relative to adjacent teeth.

Radiographic evaluation (Figure 6) at one-year post-treatment showed intact periradicular bone with no evidence of periapical pathology, no apparent progression of the replacement resorption defect, and adequate maintenance of root canal obturation.

**Figure 6:**
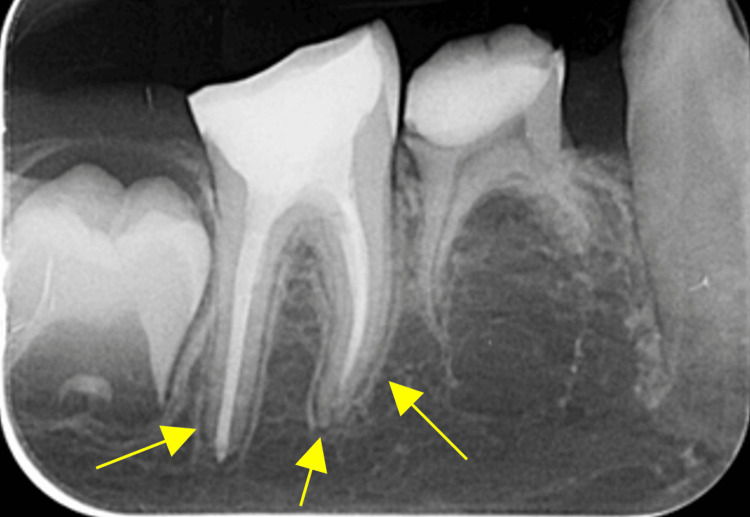
Follow-up periapical radiograph of tooth 46 with no evidence of periapical pathology or progression of the replacement resorption

The parent was counselled regarding the importance of continued long-term follow-up appointments to monitor for potential progression of the resorptive process, development of infraocclusion, and overall tooth prognosis.

## Discussion

This clinical case presents several significant features, such as the unusual occurrence of replacement resorption in a permanent molar without documented trauma, the association with multiple tooth agenesis, the diagnostic challenges encountered, the role of CBCT in treatment planning, and the successful short-term outcome of conservative management despite the poor long-term prognosis.

Although replacement resorption is frequently a late complication of dental trauma, in the case of this eight-year-old boy, the father did not mention such an event. It is possible, however, that this occurred without their knowledge. It is unlikely that occlusal trauma would cause this type of resorption [14]. Another hypothesis is that this type of resorption may have causes other than trauma. Given that the child's father presented with a cleft lip, a genetic condition, it is possible that there is a genetic predisposition for the resorptive process [15].

The furcation area region, where the resorption was in this case, experiences biomechanical forces and has limited blood supply compared to other root areas, potentially making it more susceptible to localized periodontal ligament necrosis even from minor insults [16].

The diagnostic challenges encountered in this case underscore the critical importance of CBCT in complex endodontic scenarios. Although we had intended not to perform preoperative scanning, considering the radiation dose at such a young age, the difficulties encountered in determining working length made this necessary. CBCT revealed the exact location of the radicular defect and its extension and was crucial in estimating working length and evaluating the curvatures of the mesial canals, particularly in the frontal plane. All these aspects could not be appreciated on panoramic or periapical radiography [11].

The apex locator indicating proximity to periodontium at mid-root level proved to be a crucial diagnostic clue. When the replacement resorption defect had sufficiently thinned the dentin separating the canal from the resorbed area, the apex locator detected this communication [17].

Under typical circumstances, replacement resorption in a permanent molar might warrant extraction, particularly given the high likelihood of progressive infraocclusion and eventual tooth loss [8,9]. However, in this case, the panoramic radiograph was an essential factor in the decision for conservative treatment [12], given that it revealed multiple agenesis. Preservation of tooth 46, even temporarily, prevents tilting of adjacent teeth, preserves alveolar bone volume through continued functional loading, and provides occlusal function [15], until the age when implant-supported prosthetic solutions become viable [18].

After careful study of the case, we determined that endodontic treatment could be safely performed by adapting endodontic principles and protocols. We used flexible martensitic nickel-titanium endodontic files, with diameter and taper to ensure minimal shaping in the curved mesial canals with communication to the periodontium [19]. Low concentrations of hypochlorite (2% rather than 5.25%) delivered under negative pressure to prevent extrusion and tissue toxicity [19]. The mesial canals were obturated with bioceramic materials, which offer several notable advantages over gutta-percha, such as superior sealing ability, bioactive properties that promote healing and hard tissue formation, dimensional stability, and the ability to set in a moist environment [20]. Customizing a flexible plugger from a modified ROTATE instrument demonstrated the importance of technical adaptation when conventional instruments are inadequate for specific clinical scenarios.

The one-year follow-up showing no progression of the resorptive process and the absence of infraocclusion is encouraging but must be interpreted cautiously. The natural history of replacement resorption is highly variable, with some cases showing rapid progression, while others remain stable for extended periods [1]. Due to its aggressive and asymptomatic nature of progression, long-term monitoring is essential, with clinical and radiographic evaluations recommended at six-month intervals [2].

## Conclusions

This case report describes the successful conservative management of replacement resorption in a permanent first molar in an eight-year-old child with multiple tooth agenesis and no trauma history. Key principles included early recognition, advanced imaging (CBCT) essential for complete diagnosis, and individualized treatment. Despite a guarded prognosis, conservative management may be justified when extraction would compromise or complicate future rehabilitation. In complex cases, technical adaptations enhance success, and long-term monitoring is mandatory. Continued follow-up every 6-12 months is recommended until skeletal maturity, at which point implant-supported restoration may be considered if tooth loss occurs. Conservative endodontic management can provide valuable temporizing treatment in complex pediatric cases, preserving function during critical developmental years.
